# Pharmacy Accessibility and Social Vulnerability

**DOI:** 10.1001/jamanetworkopen.2024.29755

**Published:** 2024-08-23

**Authors:** Giovanni Catalano, Muhammad Muntazir Mehdi Khan, Odysseas P. Chatzipanagiotou, Timothy M. Pawlik

**Affiliations:** 1 Department of Surgery, The Ohio State University, Wexner Medical Center and James Comprehensive Cancer Center, Columbus

## Abstract

This cross-sectional study examines the association of availability of primary care practitioners and level of socioeconomic vulnerability with risk of pharmacy deserts in regions of the US.

## Introduction

Retail pharmacy chains have been closing thousands of locations throughout the US, possibly playing a role in health care gaps.^1^ Similar to the concept of food deserts, areas in which medications are harder to obtain have been deemed pharmacy deserts.^2^ In this study, we defined how pharmacy deserts may disproportionately affect individuals living in US regions with low practitioner supply and high social vulnerability.

## Methods

Data through 2020 on communities located 10 or more miles from the nearest retail pharmacy (ie, pharmacy deserts) were sourced from TelePharm Map.^3^ Counties were stratified as high pharmacy desert density if the number of pharmacy deserts per 1000 inhabitants was in the 80th percentile or higher. Social vulnerability index (SVI) and health care practitioner data were obtained from Agency for Toxic Substances and Disease Registry^4^ and Area Health Resources File,^5^ respectively. Primary care practitioner (PCP; including family medicine, general practice, general internal medicine, general pediatrics physicians) density was calculated as the number of PCP per 10 000 inhabitants. In accordance with the Common Rule, this cross-sectional study was exempt from ethics review and informed consent requirement because only public county-level data were used. We followed the STROBE reporting guideline.

Wilcoxon rank sum test, χ^2^ test, or logistic regression analysis were used to identify associations between variables of interest. Two-sided *P* < .05 was considered statistical significance. Data analysis was performed from January to March 2024 using R 4.3.2 (R Core Team).

## Results

Among 3143 counties, 1447 (46%) had at least 1 pharmacy desert, of which 818 (56.5%) were categorized as having low and 629 (43.5%) as having high pharmacy desert density, respectively (Table). Counties with a high vs low pharmacy desert density had a higher SVI (high SVI: 238 [38.0%] vs 294 [36.0%]; low SVI: 194 [31.0%] vs 246 [30.0%]; *P* = .006) (Figure). Areas with a high pharmacy desert density had lower median [IQR] PCP density (3.65 [1.12-5.96]) vs regions with low (5.01 [3.21-7.53]) or no pharmacy (4.86 [3.10-7.40) desert density (*P* < .001). On multivariate analysis, after controlling for age and sex, both high SVI (odds ratio [OR], 1.35; 95% CI, 1.07-1.70; *P* = .01) and low PCP density (OR, 2.27; 95% CI, 1.80-2.86; *P* < .001) were associated with a higher likelihood for a county to have a high pharmacy desert density.

**Table.** County Characteristics Stratified by County-Level Pharmacy Desert Density

Characteristic	Median (IQR)				*P* value
	Overall (N = 3143)	Pharmacy desert density			
		High (n = 629)	Low (n = 818)	No pharmacy deserts (n = 1696)	
SVI, No. (%)					
Low	1037 (33)	194 (31)	246 (30)	597 (35)	.006
Moderate	1048 (33)	197 (31)	278 (34)	573 (34)	
High	1058 (34)	238 (38)	294 (36)	526 (31)	
PCP density	4.66 (2.79-7.20)	3.65 (1.12-5.96)	5.01 (3.21-7.53)	4.86 (3.10-7.40)	<.001
Total population	25 698 (10 831-67 945)	6137 (3258-10 876)	33 506 (18 011-65 970)	38 812 (16 756-112 427)	<.001
Sex					
Female, %	50.2 (48.9-51.5)	49.6 (47.9-51.3)	50.1 (48.8-51.3)	50.4 (49.4-51.6)	<.001
Male, %	49.8 (48.5- 51.1)	50.4 (48.7- 52.1)	49.9 (48.7-51.2)	49.6 (48.4-50.6)	<.001
Age >65 y, %	19.8 (17.2-22.6)	22.6 (19.7-25.8)	19.3 (16.8-21.7)	19.2 (16.9-21.8)	<.001

Abbreviation: PCP, primary care practitioner (including family medicine, general practice, general internal medicine, general pediatrics physicians); SVI, Social Vulnerability Index.

**Figure.**
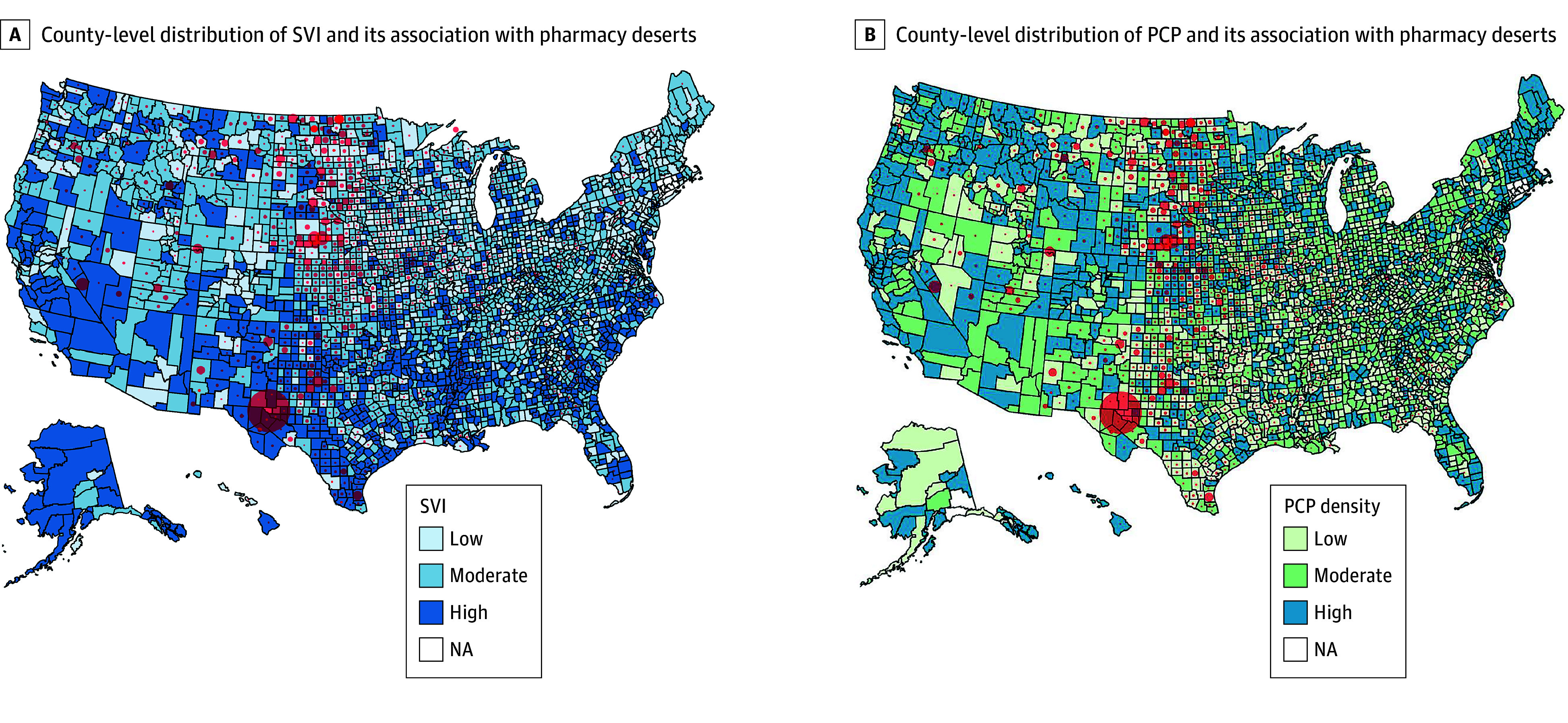
County-Level Distribution of Social Vulnerability Index (SVI) and Primary Care Practitioner (PCP) and Their Association With Pharmacy Deserts The size of the red circles represents the number of pharmacy deserts per 1000 inhabitants. The biggest circle is about 10 pharmacy deserts per 1000 inhabitants, while the smallest is about 1 per 1000 inhabitants. NA indicates not available.

## Discussion

In the US, CVS announced plans to close 900 stores in the next 3 years, and Rite Aid filed for bankruptcy.^1^ As pharmacies close, more and more individuals are left without easy access to medications, with disproportionate consequences for certain communities. Patients in higher SVI counties with a lower PCP density had a 30% to 40% higher likelihood to reside in regions with pharmacy deserts. These findings highlight how disparities compound to create barriers to access basic health care.

There is an association between SVI and number of chronic conditions. For example, diabetes and hypertension tend to be more prevalent among Black patients living in rural areas.^6^ Poor access to pharmacies is often associated with lower medication adherence. Patients in socially vulnerable communities may lack the means to travel to other pharmacies or may have limited access to broadband internet to find telepharmacy options. Furthermore, pharmacies often offer diagnostic, preventive, and emergency services. As high pharmacy desert density counties also have a lower PCP density, patients residing in these regions face increased barriers to accessing primary health care needs.

In future studies, weighted regression and inverse probability weighting could provide more insights into disparities in health care access. While the cross-sectional design limited the ability to draw causal inferences, the study demonstrated that high SVI and low PCP density were associated with concomitant risk of a pharmacy desert. This finding suggests that people already at highest risk of being neglected by the health care system are most likely to be affected by pharmacy closures. More efforts are needed to maintain access to pharmacies in underserved communities.

## Notes

Supplement.
Data Sharing Statement
