# Supplementary material for: Pharmacy Accessibility and Social Vulnerability
Source: JAMA Netw Open. 2024 Aug 23;7(8):e2429755. doi: 10.1001/jamanetworkopen.2024.29755 (PMC11344234; doi:10.1001/jamanetworkopen.2024.29755)
Supplement: Supplement. — Data Sharing Statement [file jamanetwopen-e2429755-s001.pdf]

Data Sharing Statement

Catalano. Pharmacy Accessibility and Social Vulnerability. *JAMA Netw Open*. Published August 23, 2024. doi:10.1001/jamanetworkopen.2024.29755

Data
Data available: No
